# Supplementary figures and images for: Potential of psychrotolerant rhizobacteria for the growth promotion of wheat (Triticum aestivum L.)
Source: PeerJ. 2023 Nov 30;11:e16399. doi: 10.7717/peerj.16399 (PMC10693821; doi:10.7717/peerj.16399)

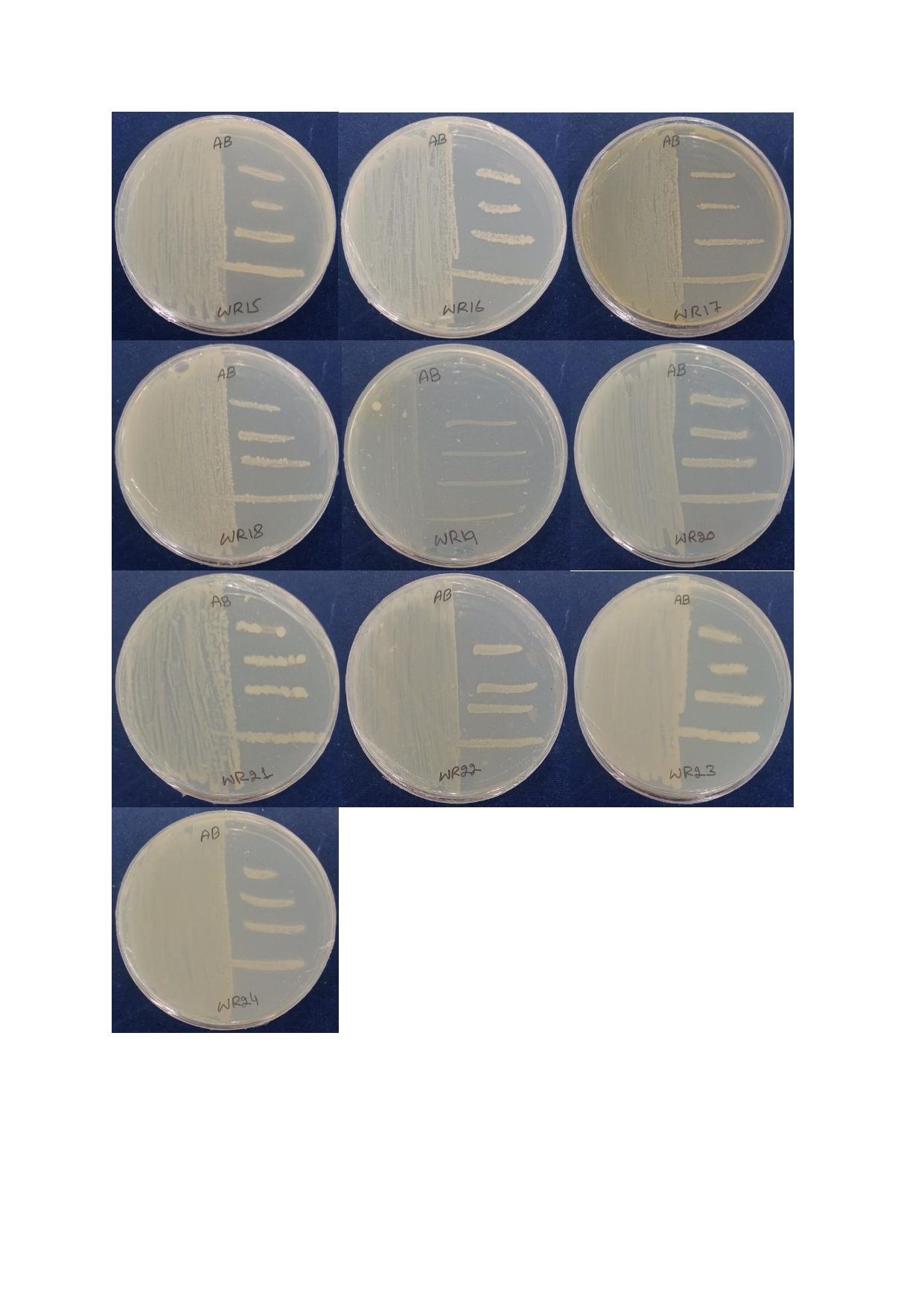

Supplement: Supplemental Information 1 — Each bacteria was streaked on a single plate. [file peerj-11-16399-s001.jpg]
